# Functional identification of DNA demethylase gene *CaROS1* in pepper (*Capsicum annuum* L.) involved in salt stress

**DOI:** 10.3389/fpls.2024.1396902

**Published:** 2024-05-01

**Authors:** Xuelian Ou, Qingzhu Hua, Jichi Dong, Kexian Guo, Minghua Wu, Yinjun Deng, Zhiming Wu

**Affiliations:** College of Horticulture and Landscape Architecture, Zhongkai University of Agriculture and Engineering, Guangzhou, China

**Keywords:** pepper, CaROS1, virus-induced gene silencing, salt stress, overexpression

## Abstract

Pepper, which is a widely cultivated important vegetable, is sensitive to salt stress, and the continuous intensification of soil salinization has affected pepper production worldwide. However, genes confer to salt tolerance are rarely been cloned in pepper. Since the REPRESSOR OF SILENCING 1 (ROS1) is a DNA demethylase that plays a crucial regulatory role in plants in response to various abiotic stresses, including salt stress. We cloned a *ROS1* gene in pepper, named *CaROS1* (LOC107843637). Bioinformatic analysis showed that the CaROS1 protein contains the HhH-GPD glycosylase and RRM_DME domains. qRT-PCR analyses showed that the CaROS1 was highly expressed in young and mature fruits of pepper and rapidly induced by salt stress. Functional characterization of the *CaROS1* was performed by gene silencing in pepper and overexpressing in tobacco, revealed that the *CaROS1* positively regulates salt tolerance ability. More detailly, *CaROS1*-silenced pepper were more sensitive to salt stress, and their ROS levels, relative conductivity, and malondialdehyde content were significantly higher in leaves than those of the control plants. Besides, *CaROS1*-overexpressing tobacco plants were more tolerant to salt stress, with a higher relative water content, total chlorophyll content, and antioxidant enzyme activity in leaves compared to those of WT plants during salt stress. These results revealed the *CaROS1* dose play a role in salt stress response, providing the theoretical basis for salt tolerance genetic engineering breeding in pepper.

## 1 Introduction

Soil salinization has been recognized as a serious environmental problem for world agriculture (Van Zelm et al., 2020; Ashraf and Munns, 2022). Salt at the bottom of the soil rises to the surface along with capillary moisture, leading to salt accumulation in the surface soil, thus causing plants to suffer from high osmotic potential. Salt stress hinders plant growth and development through ionic stress, osmotic stress, and oxidative stress (Zörb et al., 2019; Zulfiqar and Ashraf, 2021). Plants can usually adapt and maintain a balance of reactive oxygen species (ROS) under low concentrations of salt stress (Baxter et al., 2014). However, a high salt concentration leads to the excessive accumulation of ROS, which causes nucleic acid mutations and protein denaturation, leading to metabolic dysfunction and cell apoptosis (Dhindsa et al., 1981; Gill and Tuteja, 2010; Das and Roychoudhury, 2014). In order to reduce damage, plants have evolved a set of osmotic regulation, ion balance, and antioxidant defense mechanisms to enhance their salt stress tolerance (Liu et al., 2022).

5-DNA methylation is a stable and conserved epigenetic mark that occurs on the fifth carbon atom of cytosine (Vanyushin and Ashapkin, 2011) and has been implicated in genomic imprinting (Macdonald, 2012), transposable element repression (Xie et al., 2013), X-chromosome inactivation (Bala Tannan et al., 2014), gene silencing (Fu et al., 2013), and other growth and development processes. DNA methylation is usually present in symmetric CG sequences in mammals, but also occurs in asymmetric CHH sequences (where H is A, C, or T) and symmetrical CG or CHG sequences in plants (Law and Jacobsen, 2010; He et al., 2011). These modifications are mainly carried out by methyltransferases, chromomethyltransferases, and domain-rearranged methyltransferases (Liu et al., 2018). DNA methylation can be triggered by environmental changes, which prompts the opposite functions of DNA demethylase and methyltransferase to regulate DNA methylation levels (Cao et al., 2014).

Under abiotic stress, DNA methylation and demethylation work together to regulate DNA methylation levels of key genes (oxidoreductase and regulating ion balance), resulting in changes in gene expression, so as to regulate plant tolerance to stress at the physiological level (Bharti et al., 2015; Ferreira et al., 2015). REPRESSOR OF SILENCING 1 (ROS1) is a DNA demethylase whose demethylation activity is involved in base excision DNA repair and protects the genome from harmful methylation during plant development (Gong et al., 2002; Morales-Ruiz et al., 2006; Parrilla-Doblas et al., 2019; Khouider et al., 2021). Studies have shown that the products of the *ROS1* gene contribute to the regulation of plant flowering and fruit development (Lang and Gong, 2018; Liu et al., 2018). Moreover, *ROS1* gene is closely associated with the plant response to abiotic stresses. For example, the DNA methylation in the promoter region of genes associated with antioxidant pathways is reduced under salt stress in tobacco plants overexpressing *AtROS1*, leading to an increase in gene expression levels of antioxidant enzymes (such as glutathione S-transferase, ascorbate peroxidase, glutathione peroxidase and glutathione reductase) and enhanced ROS removal capacity, thereby enhancing salt tolerance (Bharti et al., 2015). Gahlaut et al. found differences in the expression of wheat dMTase proteins (ROS1 and DEMETER-Like) in different developmental stages, tissues, and varieties under heat stress (Gahlaut et al., 2022). The expression of the DNA demethylase *HcROS1* was increased, the level of genomic DNA methylation was decreased, and the plants’ salt tolerance was enhanced (Du et al., 2017). Under cold stress, Arabidopsis *ROS1* mediated DNA demethylation plays important roles in the transcription of defense genes and stress response genes (Yang et al., 2022). Sun et al. found that *ZmROS1* helps to regulate methylation in maize under salt stress (Sun et al., 2017). The overexpression of the products of the gene *NtROS2a* in tobacco could increase plants’ tolerance to abiotic stress, similar to the *Arabidopsis ROS1* gene (In Hye et al., 2015).

Pepper (*Capsicum annuum* L.) is a *Solanaceous* vegetable originating from Central and South America, and it is an industrial raw material for medicine, spicy condiments, and cosmetics (Zou and Zhu, 2022). China is the country that produces and consumes the most chili peppers in the world (Zhang et al., 2023). Salt stress is one of the important reasons for pepper yield loss and it adversely affects pepper growth and development. Although the function of the demethylase ROS1 has been investigated in many species, it has not been isolated and its function remain unclear in pepper. Therefore, we characterized the biological effects of *CaROS1* genes on the response to salt stress in pepper by gene silencing and overexpressing technologies. These results help us to understand the function of *CaROS1* and provide a theoretical basis for cultivating salt-tolerant varieties in pepper

## 2 Results

### 2.1 Identification and bioinformatics analysis of CaROS1

**Figure 1:**
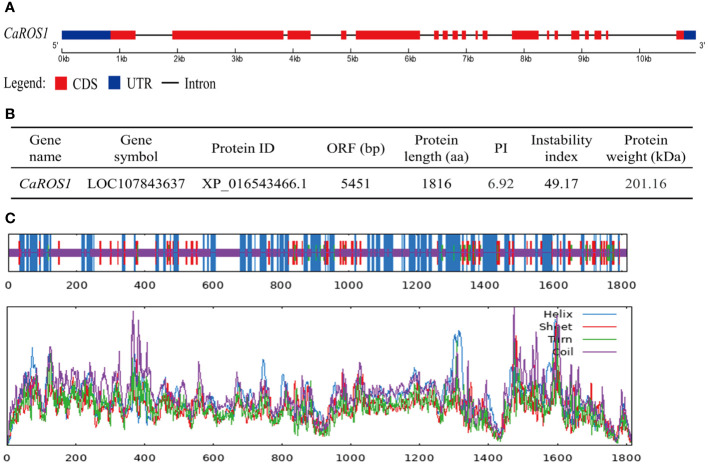
Basic information for CaROS1 in pepper. **(A)** Structure of *CaROS1* gene. Red rectangles and black lines indicate exons and introns, respectively; blue rectangles indicate the upstream and downstream sequence of *CaROS1*. **(B)** Physicochemical properties of CaROS1. **(C)** The predicted secondary structure of CaROS1.

*CaROS1* (LOC107843637), which consists of 19 exons and 18 introns, is located on chromosome 10 of pepper genome (**Figure 1A**). The length of *CaROS1* coding sequence is 5,451 bp, and the *CaROS1* gene encoding a 201.16 kDa protein containing 1,816 amino acids, with a PI of 6.92. The instability index of CaROS1 is 49.17, which means that it is considered an unstable protein (**Figure 1B**). The maximum hydropathicity value of CaROS1 is 1.83, and the minimum is -3.79 (**Supplementary Figure S1A**), thus, it is a hydrophilic protein. The CaROS1 protein has neither an obvious signal peptide nor a transmembrane domain (**Supplementary Figures S1B, C**). The secondary structure of CaROS1 comprises alpha helices (30.40%), beta turns (2.92%), extended strands (9.97%), and random coils (56.72%) (**Figure 1C**). Based on the secondary structure, the tertiary structure of CaROS1 is shown in **Supplementary Figure S1D**.

### 2.2 Phylogeny and domain analysis of ROS1

**Figure 2:**
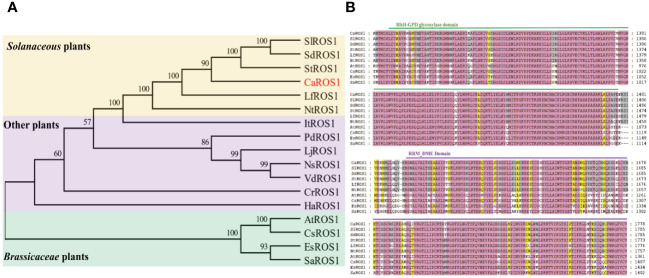
Evolutionary tree and multiple sequence alignment of ROS1 proteins. **(A)** Evolutionary tree analysis of ROS1 proteins. Red font represents the ROS1 protein in pepper. A yellow background indicates *Solanaceae* plants, a purple background indicates other plants, and a green background indicates *Brassicaceae* plants. Si (*Solanum lycopersicum* L.); Sd (*Solanum dulcamara* L.); St (*Solanum tuberosum* L.); Lf (*Lycium ferocissimum*); Nt (*Nicotiana tabacum* L.); It (*Ipomoea triloba*); Pd (*Prunus domestica* L.); Lj (*Lonicera japonica*); Ns (*Nyssa sinensis*); Vd (*Vaccinium darrowii*); Cr (*Catharanthus roseus*); Ha (*Helianthus annuus* L.); At (*Arabidopsis thaliana*); Cs (*Camelina sativa* L.); Es (*Eutrema salsugineum*); Sa (*Sinapis alba* L.). **(B)** Alignment of the ROS1 sequences of the *Brassicaceae* and *Solanaceae* families. Green lines represent the HhH-GPD domain, and purple lines represent the RRM_DME domain.

The alignment of ROS1 proteins between pepper and other species using MEGA-X software showed that the ROS1 proteins in *Solanaceous* plants (such as tomato, potato, wolfberry and tobacco) have the closer evolutionary relationships to those of in pepper (**Figure 2A**). However, the evolutionary relationships between the ROS1 proteins of *Solanaceae* and *Cruciferae* plants is relatively distant. Then, a multiple sequence alignment of the ROS1 proteins among *Solanaceae* and *Cruciferae* plants, including *Arabidopsi*s, flax, *Eutrema* and white mustard, was performed (**Figure 2B**). The results show that all ROS1 proteins contain a highly conserved HhH-GPD glycosylase domain and a RRM_DME domain. The HhH-GPD domain has a helix-hairpin-helix (HhH) motif, a Gly/Pro rich loop followed by a conserved aspartate (GPD), and a C terminal 4Fe-4S cluster. The RRM_DME structure is a predicted folded domain of an RRM-like glycolyase, which is important for RNA regulation and promoting the interaction of catalytic domain with ssDNA.

### 2.3 Subcellular localization and expression patterns of CaROS1

**Figure 3:**
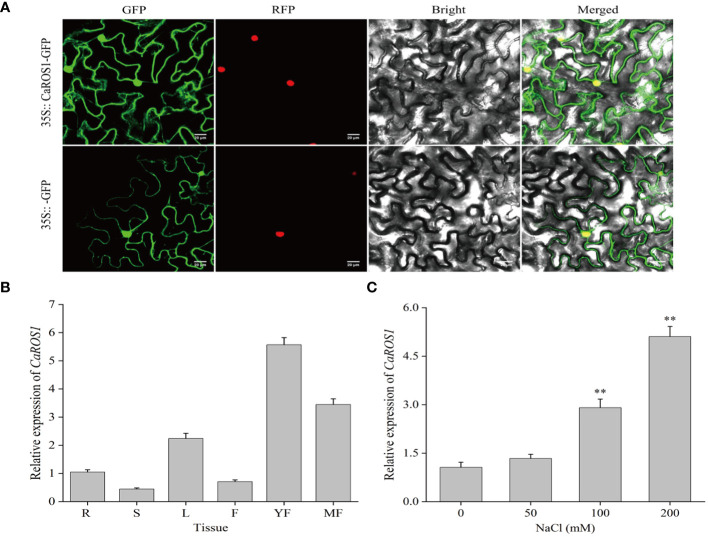
Subcellular localization and expression analysis of CaROS1. **(A)** The 35S::GFP and 35S::CaROS1 plasmids were separately transformed into tobacco leaves for subcellular localization analysis. GFP is shown in green, and nuclear markers in red. Scale bar = 20 μm. **(B)** Analysis of the expression patterns of *CaROS1* in pepper; root (R), stem (S), leaf (L), flower (F), young fruit (YF), and mature fruit (MF). The expression level of *CaROS1* in the root was set to 1. **(C)** Leaves were treated with different concentrations of NaCl (0, 50, 100, and 200 mM) for 20 days, and then the expression pattern of *CaROS1* under salt stress was determined using qRT-PCR. “**” indicates *P* < 0.01.

To examine the subcellular localization of CaROS1, the 35S::CaROS1-GFP construct was transiently expressed in tobacco leaves. The fluorescence signal of CaROS1 was detected in both the nucleus and cytoplasm of tobacco epidermal cells. This is consistent with the fluorescence signal detected for the control GFP (**Figure 3A**).

To investigate the expression patterns of *CaROS1*, its relative expression levels in different tissues, including roots, stems, leaves, flowers, young fruits, and mature fruits, were calculate based on qRT-PCR. As shown in **Figure 3B**, *CaROS1* was highly expressed in young fruits, followed by mature fruits, leaves, and roots, with the lowest expression in stems. In addition, after treated with different NaCl concentrations (0 mM, 50 mM, 100 mM, and 200 mM), we found that the expression of *CaROS1* was significantly increased in the 100 mM and 200 mM NaCl treatment groups (**Figure 3C**).

### 2.4 Influence of *CaROS1* silencing on salt tolerance in pepper

The expression of *CaROS1* was induced by salt stress (**Figure 3C**), indicating that *CaROS1* was involved in salt stress response regulation. Therefore, virus-induced gene silencing (VIGS) was used to explore the effect of *CaROS1* silencing on salt tolerance in pepper. After 25 days of transient transformation, leaves of TRV: 00 (control plants) and TRV: *CaROS1* plants were collected, and the *CaROS1* gene expression levels were determined using qRT-PCR. The *CaROS1* gene expression in gene silenced plants was 77.3% lower than that in the WT plants (**Supplementary Figure S2**).

**Figure 4:**
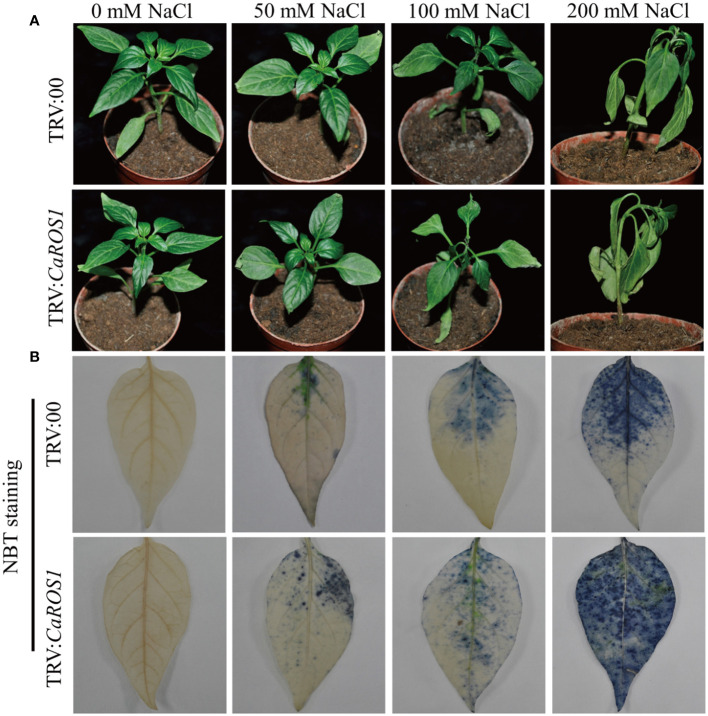
Phenotypes of TRV: 00 and TRV: CaROS1 plants after 20 days of salt stress. **(A)** Phenotypes of silenced and control plants after treatment with the indicated concentrations of salt. **(B)** Nitroblue tetrazolium staining of TRV: 00 and TRV: *CaROS1* leaves after salt stress.

The phenotypes of TRV: 00 and TRV: *CaROS1* were not significantly different under normal conditions, but a curling tip was found in parts of the control plants and *CaROS1*-silenced plants under 50 mM NaCl treatment. After the 100 mM NaCl treatment, the leaves of TRV: *CaROS1* plants were slightly withered, while only parts of the TRV: 00 plants were curled. The *CaROS1* gene silenced plants showed obvious symptoms of wilt and sagging, and the degree of damage was significantly greater than that observed for the control plants under the 200 mM NaCl treatment (**Figure 4A**).

**Figure 5:**
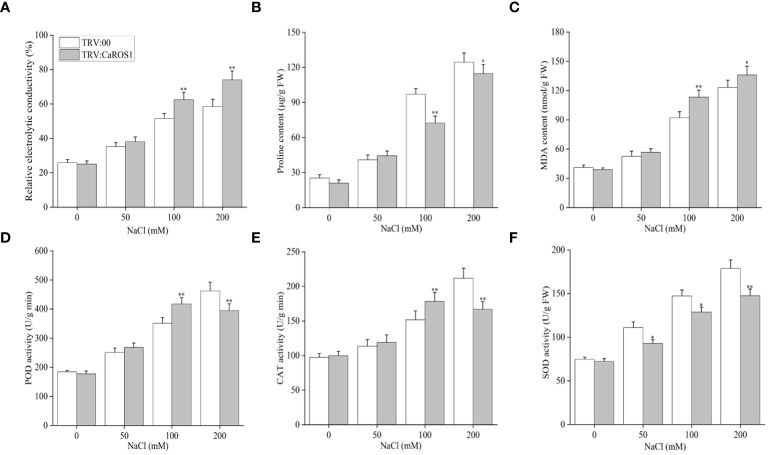
Effect of *CaROS1* silencing on salt tolerance in pepper. **(A)** Relative electrolyte conductivity, **(B)** Proline content **(C)** MDA content, **(D)** POD activity, **(E)** CAT activity **(F)** SOD activity. “*” indicates *P* < 0.05 between WT plants and *CaROS1*-silenced plants; “**” indicates *P* < 0.01.

Nitroblue tetrazolium (NBT) staining was used to detect the accumulation of H_2_O_2_ and O^2-^ in the plants. The leaves of the silenced plants showed more blue-stained spots than those of the control plants under NaCl stress (**Figure 4B**). After treatment with 100 mM and 200 mM NaCl, the relative electrolyte content (REC) and malondialdehyde (MDA) content of TRV: *CaROS1* plants were significantly higher than TRV: 00 plants (**Figures 5A, C**). Compared to that in the TRV: 00 plants, the content of proline (Pro) in the TRV: *CaROS1* plants was significantly increased (**Figure 5B**).

Salt stress can induce ROS production, so it is important to explore the activity of peroxidase (POD), catalase (CAT), and superoxide dismutase (SOD) in removing excess ROS. The POD and CAT activities in *CaROS1*-silenced plants were significantly higher than those in the TRV:00 plants after the 100 mM NaCl treatment, but significantly lower than those in the control plants after the 200 mM NaCl treatment (**Figures 5D, E**). Furthermore, the SOD activity of TRV: *CaROS1* plants was significantly lower than that in the control plants under NaCl treatment (**Figure 5F**).

### 2.5 Resistance of transgenic *CaROS1* tobacco seedlings to salt

**Figure 6:**
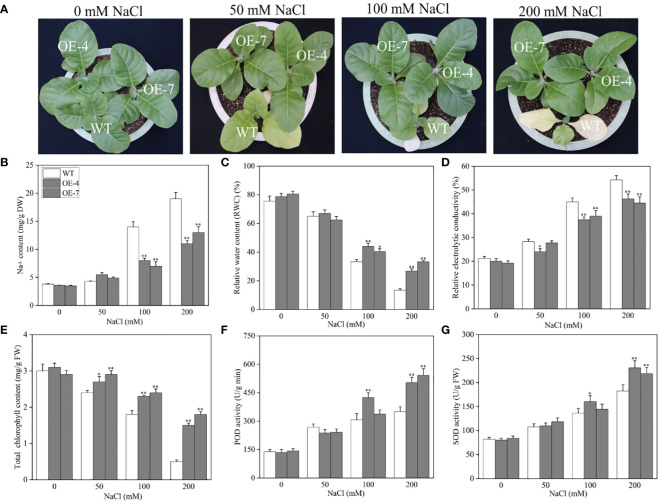
Effect of *CaROS1* overexpression on salinity stress tolerance in tobacco. **(A)** Phenotype of WT and OE lines after salt stress, **(B)** Na^+^ content, **(C)** relative water content, **(D)** relative electrolyte conductivity, **(E)** total chlorophyll content, **(F)** POD activity, and **(G)** SOD activity. “*” indicates *P* < 0.05 between WT and transgenic tobacco; “**” indicates *P* < 0.01.

To further validate the mechanism underlying the *CaROS1* response to salt stress, we produced ‘k326’ tobacco plants with *CaROS1* gene overexpression (**Supplementary Figure S3**). After analyze the expression of the *CaROS1* in different transgenic tobacco lines, the overexpression-4 (OE-4) and OE-7 lines were selected for subsequent experiments because of their high *CaROS1* expression (**Supplementary Figure S4B**). Twenty-day-old wild-type (WT) and OE lines were treated with 0, 50, 100, and 200 mM NaCl salt for 22 days. There was no significant phenotypic difference between the OE lines and the WT plants under normal conditions (**Figure 6A**). However, the leaves of the WT plants showed obvious chlorosis and yellowing, while the growth of the OE lines was less affected by salt stress. Moreover, the Na^+^ and relative electrolyte contents of the WT plants were significantly increased compared to those of the OE lines under the 100 and 200 mM NaCl treatments (**Figures 6B, D**). In contrast, the relative water content (RWC) and total chlorophyll content in the WT plants were significantly decreased compared to those in the OE lines (**Figures 6C, E**). There was no significant difference in SOD and POD activity between the WT plants and OE plants under normal and low-salt (50 mM NaCl) conditions. After the 200 mM NaCl treatment, the SOD and POD activities of the OE lines were significantly increased compared to those of the WT plants. These results indicate that the increased salt tolerance is related to the overexpression of *CaROS1* in transgenic tobacco.

## 3 Discussion

ROS1 is a demethylase that plays an important role in regulating plant growth and development (Ono et al., 2012; Zhu et al., 2018). Moreover, it is involved in plants’ responses to abiotic stress (Kim et al., 2019; Yang et al., 2022). At present, the gene encoding *ROS1* has been cloned in many species, such as *Arabidopsis* (Agius et al., 2006), maize (Tollot et al., 2016), petunia (Schwinn et al., 2014), tomato (Giordano et al., 2022), poplar (Movahedi et al., 2018), rice (Lang and Gong, 2018), cotton (Yang et al., 2019), wheat (Gahlaut et al., 2022), apple, etc (Yu et al., 2022). However, the function of *ROS1* gene in pepper remains unknown. We identified and cloned a *ROS1* gene (*CaROS1*) from pepper and found it encodes a protein containing the highly conserved HhH-GPD glycosylase and RRM_DME domains (**Figure 2B**). Phylogenetic analysis showed that CaROS1 protein has a closer evolutionary relationships with the ROS1 of *Solanaceae* (**Figure 2A**).

Subcellular localization helps us to understand the function of genes. It was found that *ROS5* in *Arabidopsis* is located in nucleus, functions as a nuclear transporter, and is required to prevent gene hypermethylation (Zhao et al., 2014). Gong et al (Gong et al., 2002). observed *AtROS1* is located in nucleus, and it has regulatory effects on gene expression and epigenetics triggered by smRNAs. We revealed that *CaROS1* is located in the cytoplasm and nucleus (**Figure 3A**). This implies that *CaROS1* not only plays a role in regulating transcription, but also associated with the function of cytoplasmic transporters. This result also suggests that the ROS1 homologs in different species may show inconsistent subcellular localization.

Previous studies have investigated the expression characteristics of *ROS1* in different tissues. Liu et al (In Hye et al., 2015). reported that the *OsROS1* in rice is highly expressed in mature pollen, anthers, and the ovary, but rarely expressed in roots and endosperm. Xu et al (Xu, 2023). reported that *ZmROS1a* and *ZmROS1b* in maize are highly expressed in embryo and endosperm. In this study, we found *CaROS1* is highly expressed in young fruits of pepper, followed by mature fruits and flowers, and has the lowest expression in the stem (**Figure 3B**). These results suggest that *CaROS1* gene may be involved in the regulation of floral organ development and fruit ripening. Furthermore, *CaROS1* gene expression increased with increasing NaCl concentration under salt stress (**Figure 3C**). This result suggests that there is a positive relationship between the expression of *CaROS1* and the salt stress dose response in pepper seedlings.

VIGS is a gene transcriptional repression technique used to study gene function in plants. It is used as a reverse genetic tool for the post-transcriptional silencing of a specific gene before phenotypic identification and gene expression in order to determine the roles of target genes in plant growth and development (Ramegowda et al., 2014). The *CaROS1* gene was silenced in pepper using VIGS to explore the role of this gene in the salt stress response. The results showed that the TRV: *CaROS1* plants were more sensitive to salt stress than the TRV: 00 plants (**Figure 4A**). ROS include hydrogen peroxide (H_2_O_2_), superoxide (O^2-^), and hydroxyl radical (OH^-^), which are byproducts of aerobic metabolism (Mansour and Salama, 2019). Under salt stress, a high accumulation of ROS causes the degeneration of cellular proteins, leading to enzyme inactivation, nucleic acid degradation, cell membrane damage, carbohydrate oxidation, and ultimately cell death (Mansour and Salama, 2020; Mansour and Hassan, 2022). In our study, we found that the proline content of *CaROS1*-silenced plants was lower than that of the control plants under salt stress, while the MDA content showed the opposite trend (**Figures 5B, C**). There is an adaptive water retention mechanism in organisms under stress that is related to the *de novo* synthesis of compatible solutes (glycine betaine, proline, sugars, and polyamines) and ion flux across cellular membranes (Hameed et al., 2021). Under abiotic stress, these substances can make cells more tolerant, and proline is known to protect the photosynthetic apparatus in cytokinin-dependent photorespiration under salinity. Strong exogenous stress can cause an increase in proline. Thus, the severe damage in *CaROS1*-silenced plants caused by salt stress, making them unable to accumulate the large amounts of proline needed to maintain the cell’s osmotic potential (Ashraf and Foolad, 2007; Shin et al., 2020). In contrast, When *CaROS1*-silenced subjected to salt stress, superoxide free radicals are produced for the peroxidation of membrane lipids to produce a large amount of malondialdehyde. The excessive accumulation of MDA causes the cross-linking polymerization of vital macromolecules such as proteins and nucleic acids, leading to changes in the structure and function of the cell membrane (Guo et al., 2019). The accumulation of ROS induced by environmental stress causes plants to eliminate ROS through the signal transduction of the synthetic antioxidant enzyme system, which includes superoxide dismutase SOD, CAT and POD (Acet and Kadıoğlu, 2020; Wang et al., 2021). After the 50 mM and 100 mM NaCl treatments, the POD and CAT activities of TRV: *CaROS1* plants were increased compared to those of the TRV: 00 plants (**Figures 5D, E**). This is because *CaROS1* silenced plants require higher CAT and POD enzyme activity to alleviate the damage caused by low concentration of salt stress. In contrast, under 200 mM NaCl stress, the TRV: *CaROS1* plants had a lower antioxidant enzyme activity than the TRV: 00 plants (**Figures 5D, F**). These results indicate that, when salt concentrations are too high, *CaROS1*-silenced plants fail to remove large amounts of ROS, causing severe damage to the plants, indicating that the *CaROS1* gene may be critical for the survival of pepper plants in heavily salt polluted environments.

*CaROS1* gene was then overexpressed in tobacco and the transgenic plants were treated with different NaCl concentrations to further confirm the role of *CaROS1* gene in salt stress. WT plants showed more severe damage than the OE lines under salt stress. Under high salt stress, the leaves of control plants suffered severely chlorosis, and some leaves turned yellow and even withered, while the OE lines grew well (**Figure 6A**). This result is consistent with previous studies of *ROS1* under salt stress (Bharti et al., 2015). In addition, with increasing salt concentrations, the chlorophyll content decreased, while the accumulation of Na^+^ increased (**Figures 6B, E**). The toxicity of salt stress mainly arises from ion toxicity and osmotic stress (Zhang et al., 2020; Liu et al., 2022). Under salt stress, the content of Na^+^ and Cl^-^ increased in plants, which reduced the affinity between chlorophyll and chlorophyll proteins, and finally chlorophyll dissociated (Loudari et al., 2020). Moreover, severe salt stress will inhibit the activity of key enzymes in chlorophyll synthesis, activate chlorophyll enzymes and accelerate their degradation (Athar et al., 2022). When the salt concentration of soil increased, the soil’s water potential is lower than that of the plant’s root tip cells, which prevents water absorption through the plant’s roots, leading to a water shortage and osmotic stress in the plant (Lv, 2014). In this study, the relative water content in OE lines did not change significantly, however, in control plants it decreased with increasing salt treatment concentrations (**Figure 6C**). Furthermore, salt stress disrupts the balance between ROS production and clearance in plant cells, resulting in increasing ROS concentrations that activate ROS-scavenging enzymes. After 200 mM sodium chloride treatment, we found the *CaROS1* overexpressing plants had higher SOD and POD activity than WT plants (**Figures 6F, G**). The above results indicate that transgenic tobacco overexpressing *CaROS1* showed tolerance to salt stress. This is similar to previous studies on overexpression of *AtROS1* under abbiotic stress (Bharti et al., 2015; Yang et al., 2022; Bharti et al., 2023).

To summarize, we isolated the *CaROS1* gene, revealed its expression patterns, analyzed the function of *CaROS1* by gene silencing and overexpressing strategy, and investigated its possible physiological role. Overall, *CaROS1* was found to positively regulate the salt stress tolerance in pepper.

## 4 Materials and methods

### 4.1 Experimental materials and treatments

In this study, the pepper variety “zunla-1” was provided by the Vegetable Research Institute of the Guangdong Academy of Agricultural Sciences, and the “k326” tobacco was provided by the College of Horticulture and Landscape Architecture (Zhongkai College of Agricultural Engineering). Pepper seeds were disinfected with 70% ethanol and 1% sodium hypochlorite and then washed with ultrapure water and soaked for 12 h, and sprouting was accelerated in a 28 °C incubator culture. Germinated seeds were selected and sown into vegetative soil and grown in an incubator at 25 °C in light (16 h) and 20 °C in darkness (8 h) until 4-5 true leaves appeared. For the *CaROS1* tissue-specific expression analysis, six tissues, including root (R), stem (S), leaf (L), flower (F), young fruit (YF), and mature fruit (MF), were collected. For the gene-silenced pepper, samples were collected from TRV: *CaROS1* and TRV: 00 plants after 20 days of NaCl stress at 0, 50, 10, and 200 mM.

### 4.2 Bioinformatics analysis of CaROS1

The physicochemical properties of CaROS1 proteins, including their amino acids, isoelectric points (PI), instability indices, and protein weights, were analyzed using the National Center for Biotechnology Information and ExPASy platforms. Gene structure maps were drawn using GSDS. The pro/hydrophobic regions of the CaROS1 protein were analyzed using ProtScale, and the signal peptide and transmembrane domains were predicted using SignaIP 4.1 and TMHMM 2.0. The secondary and tertiary structures of the CaROS1 proteins were analyzed using NPSA-PRABI and SWISS-MODEL. Links to the bioinformatics analysis website are provided in **Supplementary Table S1**.

### 4.3 Multiple sequence alignment and phylogenetic tree analysis of ROS1 proteins

To analyze the affinity of the pepper ROS1 protein to different species, the proteins of 17 species, including pepper (XP_016543466.1), tomato (XP_004249459.3 and XP_019071586.1), potato (XP_004249459.3), barbary wolfberry (XP_059296260.1), tobacco (XP_016488674.1), sweet potato (XP_031102985.1), tupelo (KAA8516668.1), vinca (KAI5664841.1), blueberry (KAI5664841.1), sunflower (XP_022000791.1), honeysuckle (ALA55995.1), peach tree (BBH07700.1), *Arabidopsis thaliana* (NP_181190.3), flax (XP_010509466.1), *Eutrema salsugineum* (KAF8101783.1), and white mustard (XP_006410826.1), were obtained from the NCBI. Phylogenetic analysis was performed by using MEGA-X software. The adjacent connection method (Neighborjoining, NJ) was adopted to construct a phylogenetic evolutionary tree, and the calibration parameter for the boot-strap method was set to 1000. Multiple sequence alignment for the ROS1 of *Solanaceous* plants (such as tomato, potato, wolfberry, and tobacco) and that of *Cruciferae* (*Arabidopsis*, flax, *Eutrema*, and white mustard) was performed using MEGA-X and GeneDoc.

### 4.4 Expression analysis of *CaROS1* gene

Total RNA was isolated from pepper and tobacco samples using the Total RNA Kit I (OMEGA, Guangzhou, China), and the concentrations were detected using an ND-100C ultrascopic UV visible spectrophotometer (MIULAB, China). The first strand cDNA was synthesized with the Hifair® 1st Strand cDNA Synthesis Super Mix for qPCR kit (Yeasen, Shanghai, China). The qRT-PCR was performed using the 7500 Fast Real-Time PCR System (Thermo Fisher Scientific, Shanghai, China) and 2×ChamQ Universal SYBR qPCR Master Mix (Vazyme, Nanjing, China). The relative expression of *CaROS1* was analyzed using the 2^-ΔΔCt^ method (Livak and Schmittgen, 2001). The primer information for *CaROS1* is provided in **Supplementary Table S2**.

### 4.5 Subcellular localization of CaROS1

In order to construct the 35S::CaROS1-GFP fusion protein, the coding sequence of *CaROS1* was cloned into the N terminus of GFP under the control of the CaMV-35S promoter in the vector pNC-Cam1304-SubN (Chinese Academy of Tropical Agricultural Sciences, China). The 35S::CaROS1-GFP constructs were then transformed into tobacco leaves (Zhao et al., 2017). Fluorescent signals were observed and images were taken using an FV1000 confocal laser microscope (OLYMPUS, Japan). For the GFP protein and nuclear marker location observation, 488 nm and 561 nm excitation spectrum, 510 nm and 580 nm emission spectrum, was used in this experiment, respectively.

### 4.6 VIGS of pepper seedlings

The coding region of *CaROS1* was joined to the pNC-TRV2 vector (Chinese Academy of Tropical Agricultural Sciences) to form pTRV2:*CaROS1*. Then, pTRV1 (Chinese Academy of Tropical Agricultural Sciences), pTRV2:00 (control), and pTRV2:*CaROS1* were transformed into *Agrobacterium tumefaciens* strain GV3101 (WeGo, Guangzhou, China). *CaROS1* was silenced in pepper using the method described by Singh et al (Singh et al., 2022). After 25 days of silencing, leaves of TRV2:*CaROS1* plants and pTRV2:00 plants were collected to measure the silencing efficiency.

### 4.7 Construction of overexpressed *CaROS1* cloning vector

The *CaROS1* sequence was amplified by PCR using the 2×Hieff Canace® Plus PCR Master Mix (Yeasen, Shanghai, China). The purified amplified products were ligated into the pNC-Cam1304-35S vector (Chinese Academy of Tropical Agricultural Sciences) and transfected into Trans1-T1 *Phage Resistant* chemocompetent cells (WeGo, Guangzhou, China). Then, they were placed on Luria–Bertani (LB) (50 μg/mL Kan) solid medium for 12 h, and single *E. coli* colonies were expanded in LB liquid medium (37 °C, 210 rpm) to an OD 600 of 0.65. The positive clones were identified by PCR and sent to Sangon Biological Company (Shanghai, China) for sequencing confirmation.

### 4.8 Tobacco conversion

The “k326” tobacco seeds were sterilized and sown into MS (Murashige Skoog) medium until 3–4 true leaves grew. *CaROS1* transgenic tobacco was obtained using *Agrobacterium tumefaciens*-mediated leaf disc transformation (Oh et al., 2005). In addition, the lines were identified using qRT-PCR during the T1 seedling period (**Supplementary Figure S4**). T1 tobacco seeds were further screened for T2 lines on MS medium containing 50 μg/mL kanamycin. The T3 line was used for the subsequent experiments.

### 4.9 Salt-stress treatment and sample collection

Pepper seeds were soaked for 8 h, germinated in a 28 °C incubator, and seeded into vegetative soil (matrix, vermiculite, and perlite mixed at 3:1:1 ratio). Each plant was planted in an independent basin (same basins were used). Five-six true leaf stage pepper seedlings were subjected to salt stress for 0, 50, 100 and 200 mM. The 50 ml of NaCl solution was poured into the soil of each pepper plant for every four days. Samples were collected after 20 days of salt stress treatment. For the treatment of silenced plants, TRV: *CaROS1* and TRV: 00 plants were treated with 0, 50, 100 and 200 mM NaCl solution for 20 days. The pepper leaves were then harvested and all samples were immediately placed in liquid nitrogen and then stored at-80 °C.

“K326” tobacco was used for gene transformation in this experiment. Seeds of wild-type tobacco and transgenic lines were soaked in water, vernalized at 4 °C for 48 h, and then sown in a high-pressure-sterilized nutrient mixture. The tobacco was placed in a temperature incubator (26 °C light (16 h)/20 °C dark (8 h) conditions) for 20 days. Then they were placed in a big pot and watered every four days with different concentrations of NaCl solution (500 ml per basin) at room temperature. Samples were collected after 22 days of salt stress treatment.

### 4.10 NBT staining

We performed NBT staining of pepper leaves following Wohlgemuth’s method (Wohlgemuth et al., 2002). TRV2:00 and *CaROS1*-silenced pepper leaves were immersed in NBT staining solution in the dark for 12 h, then added to 50 mL of absolute ethanol and heated to decoloring at 100 °C. The concentration of the NBT staining solution was 0.5 mg/mL.

### 4.11 Determination of plant physiological indicators

We measured the REC and RWC as described by Choluj et al (Chołuj et al., 2014). and Pan et al (Pan et al., 2012), respectively. The MDA content, proline content, and activity of antioxidant enzymes (POD, SOD, and CAT) were measured using a plant detection kit (Nanjing Jiancheng Biotech, China), according to the manufacturer’s instructions. The Na^+^ content and total chlorophyll content were measured as described by Li and Arkus (Arkus et al., 2005; Li, 2015).

### 4.12 Statistical analysis

Statistical analysis was performed using SPSS Statistics 26.0. Differences were considered significant at *P* < 0.05 and extremely significant at *P* < 0.01.

## 5 Conclusions

We cloned the *CaROS1* gene, which encodes a protein that contains the highly conserved HhH-GPD and RRM _ DME domains and localizes in the nucleus. According to the qRT-PCR analysis, *CaROS1* was highly expressed in the young and mature fruit of pepper, and was rapidly induced by salt stress. We verified its biological function through the silencing and overexpression of *CaROS1* in pepper and tobacco, respectively. Compared with control plants, *CaROS1*-silenced plants showed more severe damage, lower antioxidant enzyme activity, and reduced tolerance to salt stress. The *CaROS1* transgenic tobacco showed higher relative water content and total chlorophyll content than the WT plants, and ROS accumulated less in the transgenic tobacco than in the WT plants.The above results indicate that *CaROS1* gene is valuable in regulating the salt tolerance of pepper.

## Data availability statement

The datasets presented in this study can be found in online repositories. The names of the repository/repositories and accession number(s) can be found in the article/**Supplementary Material**.

## Author contributions

XO: Data curation, Methodology, Writing – original draft. QH: Investigation, Software, Writing – review & editing. JD: Project administration, Validation, Writing – review & editing. KG: Methodology, Supervision, Validation, Writing – review & editing. MW: Investigation, Project administration, Writing – review & editing. YD: Formal Analysis, Validation, Writing – original draft. ZW: Conceptualization, Funding acquisition, Resources, Supervision, Writing – review & editing.

## References

[B1] Acet T. Kadıoğlu A. (2020). SOS5 gene-abscisic acid crosstalk and their interaction with antioxidant system in Arabidopsis thaliana under salt stress. Physiol. Mol. Biol. Plants 26, 1831–1845. doi: 10.1007/s12298-020-00873-4 32943819 PMC7468026

[B2] Agius F. Kapoor A. Zhu J. K. (2006). Role of the Arabidopsis DNA glycosylase/lyase ROS1 in active DNA demethylation. Proc. Natl. Acad. Sci. 103, 11796–11801. doi: 10.1073/pnas.0603563103 16864782 PMC1544249

[B3] Arkus K. A. J. Cahoon E. B. Jez J. M. (2005). Mechanistic analysis of wheat chlorophyllase. Arch. Biochem. Biophysics 438, 146–155. doi: 10.1016/j.abb.2005.04.019 15913540

[B4] Ashraf M. Foolad M. R. (2007). Roles of glycine betaine and proline in improving plant abiotic stress resistance. Environ. Exp. Bot. 59, 206–216. doi: 10.1016/j.envexpbot.2005.12.006

[B5] Ashraf M. Munns R. (2022). Evolution of approaches to increase the salt tolerance of crops. Crit. Rev. Plant Sci. 41, 128–160. doi: 10.1080/07352689.2022.2065136

[B6] Athar H. U. R. Zulfiqar F. Moosa A. Ashraf M. Zafar Z. U. Zhang L.. (2022). Salt stress proteins in plants: An overview. Front. Plant Sci. 13. doi: 10.3389/fpls.2022.999058 PMC9800898 36589054

[B7] Bala Tannan N. Brahmachary M. Garg P. Borel C. Alnefaie R. Watson C. T.. (2014). DNA methylation profiling in X;autosome translocations supports a role for L1 repeats in the spread of X chromosome inactivation. Hum. Mol. Genet. 23, 1224–1236. doi: 10.1093/hmg/ddt553 24186870 PMC3919006

[B8] Baxter A. Mittler R. Suzuki N. (2014). ROS as key players in plant stress signalling. J. Exp. Bot. 65, 1229–1240. doi: 10.1093/jxb/ert375 24253197

[B9] Bharti P. Mahajan M. Vishwakarma A. K. Bhardwaj J. Yadav S. K. (2015). AtROS1 overexpression provides evidence for epigenetic regulation of genes encoding enzymes of flavonoid biosynthesis and antioxidant pathways during salt stress in transgenic tobacco. J. Exp. Bot. 66, 5959–5969. doi: 10.1093/jxb/erv304 26116024 PMC4566984

[B10] Bharti P. Yadav S. K. Hallan V. (2023). Influence of AtROS1 demethylase on transcription factors involved in tobacco plant defense. J. Plant Biochem. Biotechnol. 32, 296–303. doi: 10.1007/s13562-022-00805-1

[B11] Cao D. Ju Z. Gao C. Mei X. Fu D. Zhu H.. (2014). Genome-wide identification of cytosine-5 DNA methyltransferases and demethylases in Solanum lycopersicum. Gene 550, 230–237. doi: 10.1016/j.gene.2014.08.034 25149677

[B12] Chołuj D. Wiśniewska A. Szafrański K. M. Cebula J. Gozdowski D. Podlaski S. (2014). Assessment of the physiological responses to drought in different sugar beet genotypes in connection with their genetic distance. J. Plant Physiol. 171, 1221–1230. doi: 10.1016/j.jplph.2014.04.016 25014257

[B13] Das K. Roychoudhury A. (2014). Reactive oxygen species (ROS) and response of antioxidants as ROS-scavengers during environmental stress in plants. Front. Environ. Sci. 2. doi: 10.3389/fenvs.2014.00053

[B14] Dhindsa R. S. Plumb-Dhindsa P. Thorpe T. A. (1981). Leaf senescence: correlated with increased levels of membrane permeability and lipid peroxidation, and decreased levels of superoxide dismutase and catalase. J. Exp. Bot. 32, 93–101. doi: 10.1093/jxb/32.1.93

[B15] Du C. Zhang J. Zhang L. Zhang F. (2017). Correlation analysis of DNA methylation and Expression of Demethylation Enzyme gene (ROS1) in Halostachys caspica under Salt Stress. Xinjiang Agric. Sci. 54, 878–885. doi: 10.6048/j.issn.1001-4330.2017.05.011

[B16] Ferreira L. J. Azevedo V. Maroco J. Oliveira M. M. Santos A. P. (2015). Salt tolerant and sensitive rice varieties display differential methylome flexibility under salt stress. PloS One 10, e0124060. doi: 10.1371/journal.pone.0124060 25932633 PMC4416925

[B17] Fu Y. Kawabe A. Etcheverry M. Ito T. Toyoda A. Fujiyama A.. (2013). Mobilization of a plant transposon by expression of the transposon-encoded anti-silencing factor. EMBO J. 32, 2407–17-17. doi: 10.1038/emboj.2013.169 23900287 PMC3773815

[B18] Gahlaut V. Samtani H. Gautam T. Khurana P. (2022). Identification and characterization of DNA demethylase genes and their association with thermal stress in wheat (Triticum aestivum L.). Front. Genet. 13. doi: 10.3389/fgene.2022.894020 PMC9355123 35938005

[B19] Gill S. S. Tuteja N. (2010). Reactive oxygen species and antioxidant machinery in abiotic stress tolerance in crop plants. Plant Physiol. Biochem. 48, 909–930. doi: 10.1016/j.plaphy.2010.08.016 20870416

[B20] Giordano A. Santo Domingo M. Quadrana L. Pujol M. Martín-Hernández A. M. Garcia-Mas J. (2022). CRISPR/Cas9 gene editing uncovers the roles of CONSTITUTIVE TRIPLE RESPONSE 1 and REPRESSOR OF SILENCING 1 in melon fruit ripening and epigenetic regulation. J. Exp. Bot. 73, 4022–4033. doi: 10.1093/jxb/erac148 35394503

[B21] Gong Z. Morales-Ruiz T. Ariza R. R. Roldán-Arjona T. David L. Zhu J.-K. (2002). ROS1, a repressor of transcriptional gene silencing in arabidopsis, encodes a DNA glycosylase/lyase. Cell 111, 803–814. doi: 10.1016/S0092-8674(02)01133-9 12526807

[B22] Guo H. Hu Z. Zhang H. Min W. Hou Z. (2019). Comparative effects of salt and alkali stress on antioxidant system in cotton (Gossypium hirsutum L.) leaves. Open Chem. 17, 1352–1360. doi: 10.1515/chem-2019-0147

[B23] Hameed A. Ahmed M. Z. Hussain T. Aziz I. Ahmad N. Gul B.. (2021). Effects of salinity stress on chloroplast structure and function. Cells 10, 2023. doi: 10.3390/cells10082023 34440792 PMC8395010

[B24] He X.-J. Chen T. Zhu J.-K. (2011). Regulation and function of DNA methylation in plants and animals. Cell Res. 21, 442–465. doi: 10.1038/cr.2011.23 21321601 PMC3152208

[B25] In Hye L. Jang Sun C. Marjohn N. Yong-Gu C. Kwon Kyoo K. Yu Jin J. (2015). Regulation of abiotic stress response through ntROS2a-mediated demethylation in tobacco. Plant Breed. Biotechnol. 3, 108–118. doi: 10.9787/PBB.2015.3.2.108

[B26] Khouider S. Borges F. Leblanc C. Ungru A. Schnittger A. Martienssen R.. (2021). Male fertility in Arabidopsis requires active DNA demethylation of genes that control pollen tube function. Nat. Commun. 12, 410. doi: 10.1038/s41467-020-20606-1 33462227 PMC7813888

[B27] Kim J.-S. Lim J. Y. Shin H. Kim B.-G. Yoo S.-D. Kim W. T.. (2019). ROS1-dependent DNA demethylation is required for ABA-inducible NIC3 expression. Plant Physiol. 179, 1810–1821. doi: 10.1104/pp.18.01471 30692220 PMC6446795

[B28] Lang Z. Gong Z. (2018). A role of OsROS1 in aleurone development and nutrient improvement in rice. Proc. Natl. Acad. Sci. 115, 11659–11660. doi: 10.1073/pnas.1815760115 30385630 PMC6243263

[B29] Law J. A. Jacobsen S. E. (2010). Establishing, maintaining and modifying DNA methylation patterns in plants and animals. Nat. Rev. Genet. 11, 204–220. doi: 10.1038/nrg2719 20142834 PMC3034103

[B30] Li H. (2015). The effect and mechanism of exogenous silicon on salt resistance of tomato seedlings (Shanxi, China: Northwest A&F University).

[B31] Liu C. Li H. Lin J. Wang Y. Xu X. Cheng Z.-M.. (2018). Genome-wide characterization of DNA demethylase genes and their association with salt response in pyrus. Genes 9, 398. doi: 10.3390/genes9080398 30082643 PMC6116010

[B32] Liu C. Mao B. Yuan D. Chu C. Duan M. (2022). Salt tolerance in rice: Physiological responses and molecular mechanisms. Crop J. 10, 13–25. doi: 10.1016/j.cj.2021.02.010

[B33] Liu J. Wu X. Yao X. Yu R. Larkin P. J. Liu C.-M. (2018). Mutations in the DNA demethylase OsROS1 result in a thickened aleurone and improved nutritional value in rice grains. Proc. Natl. Acad. Sci. 115, 11327–11332. doi: 10.1073/pnas.1806304115 30275307 PMC6217383

[B34] Livak K. J. Schmittgen T. D. (2001). Analysis of relative gene expression data using real-time quantitative PCR and the 2^–ΔΔCT^ method. Methods 25, 402–408. doi: 10.1006/meth.2001.1262 11846609

[B35] Loudari A. Benadis C. Naciri R. Soulaimani A. Zeroual Y. Gharous M. E.. (2020). Salt stress affects mineral nutrition in shoots and roots and chlorophyll a fluorescence of tomato plants grown in hydroponic culture. J. Plant Interact. 15, 398–405. doi: 10.1080/17429145.2020.1841842

[B36] Lv B. S. (2014). Physiological and molecular mechanisms in response to saline-alkaline stress in rice (Oryza sativa L.) (Jilin, China: Northeast Institute of Geography and Agroecology, Chinese Academy of Sciences).

[B37] Macdonald W. A. (2012). Epigenetic mechanisms of genomic imprinting: common themes in the regulation of imprinted regions in mammals, plants, and insects. Genet. Res. Int. 2012, 585024. doi: 10.1155/2012/585024 22567394 PMC3335465

[B38] Mansour M. M. F. Hassan F. A. S. (2022). How salt stress-responsive proteins regulate plant adaptation to saline conditions. Plant Mol. Biol. 108, 175–224. doi: 10.1007/s11103-021-01232-x 34964081

[B39] Mansour M. M. F. Salama K. H. A. (2019). “Cellular mechanisms of plant salt tolerance,” in Microorganisms in saline environments: strategies and functions, ed. Giri B. Varma A. (Heidelberg, Berlin: Springer) 169–210. doi: 10.1007/978-3-030-18975-4_8

[B40] Mansour M. M. F. Salama K. H. A. (2020). “Proline and abiotic stresses: responses and adaptation,” in Plant ecophysiology and adaptation under climate change: mechanisms and perspectives II: mechanisms of adaptation and stress amelioration (Springer Singapore, Singapore), 357–397.

[B41] Morales-Ruiz T. Ortega-Galisteo A. P. Ponferrada-Marín M. I. Martínez-Macías M. I. Ariza R. R. Roldán-Arjona T. (2006). DEMETER and REPRESSOR OF SILENCING 1 encode 5-methylcytosine DNA glycosylases. Proc. Natl. Acad. Sci. 103, 6853–6858. doi: 10.1073/pnas.0601109103 16624880 PMC1458983

[B42] Movahedi A. Sang M. Zhang J. Mohammadi K. Sun W. Yaghuti A. A. Z.. (2018). Functional analyses of ptROS1-RNAi in poplars and evaluation of its effect on DNA methylation. J. Plant Biol. 61, 227–240. doi: 10.1007/s12374-017-0410-7 29549759

[B43] Oh S.-K. Park J. M. Joung Y. H. Lee S. Chung E. Kim S.-Y.. (2005). A plant EPF-type zinc-finger protein, CaPIF1, involved in defence against pathogens. Mol. Plant Pathol. 6, 269–285. doi: 10.1111/j.1364-3703.2005.00284.x 20565656

[B44] Ono A. Yamaguchi K. Fukada-Tanaka S. Terada R. Mitsui T. Iida S. (2012). A null mutation of ROS1a for DNA demethylation in rice is not transmittable to progeny. Plant J. 71, 564–574. doi: 10.1111/j.1365-313X.2012.05009.x 22448681

[B45] Pan Y. Seymour G. B. Lu C. Hu Z. Chen X. Chen G. (2012). An ethylene response factor (ERF5) promoting adaptation to drought and salt tolerance in tomato. Plant Cell Rep. 31, 349–360. doi: 10.1007/s00299-011-1170-3 22038370

[B46] Parrilla-Doblas J. T. Roldán-Arjona T. Ariza R. R. Córdoba-Cañero D. (2019). Active DNA demethylation in plants. Int. J. Mol. Sci. 20, 4683. doi: 10.3390/ijms20194683 31546611 PMC6801703

[B47] Ramegowda V. Mysore K. S. Senthil-Kumar M. (2014). Virus-induced gene silencing is a versatile tool for unraveling the functional relevance of multiple abiotic-stress-responsive genes in crop plants. Front. Plant Sci. 5. doi: 10.3389/fpls.2014.00323 PMC4085877 25071806

[B48] Schwinn K. E. Boase M. R. Bradley J. M. Lewis D. H. Deroles S. C. Martin C. R.. (2014). MYB and bHLH transcription factor transgenes increase anthocyanin pigmentation in petunia and lisianthus plants, and the petunia phenotypes are strongly enhanced under field conditions. Front. Plant Sci. 5. doi: 10.3389/fpls.2014.00603 PMC4220640 25414715

[B49] Shin Y. K. Bhandari S. R. Cho M. C. Lee J. G. (2020). Evaluation of chlorophyll fluorescence parameters and proline content in tomato seedlings grown under different salt stress conditions. Horticulture Environment Biotechnol. 61, 433–443. doi: 10.1007/s13580-020-00231-z

[B50] Singh A. K. Ghosh D. Chakraborty S. (2022). “Optimization of tobacco rattle virus (TRV)-based virus-induced gene silencing (VIGS) in tomato,” in Plant gene silencing: methods and protocols (Springer US, New York, NY), 133–145. doi: 10.1007/978-1-0716-1875-2_9 35325421

[B51] Sun L. Hu K. Deng J. Miao X. Chang G. Wang X.. (2017). Bioinformatics and Gene Expression Analysis of DNA Demethylase ROS1 under Salt Stress in Maize (Zea mays). Mol. Plant Breed. 15, 3393–3400. doi: CNKI:SUN:FZZW.0.2017-09-001

[B52] Tollot M. Assmann D. Becker C. Altmüller J. Dutheil J. Y. Wegner C.-E.. (2016). The WOPR protein ros1 is a master regulator of sporogenesis and late effector gene expression in the maize pathogen ustilago maydis. PloS Pathog. 12, e1005697. doi: 10.1371/journal.ppat.1005697 27332891 PMC4917244

[B53] Van Zelm E. Zhang Y. Testerink C. (2020). Salt tolerance mechanisms of plants. Annu. Rev. Plant Biol. 71, 403–433. doi: 10.1146/annurev-arplant-050718-100005 32167791

[B54] Vanyushin B. F. Ashapkin V. V. (2011). DNA methylation in higher plants: Past, present and future. Biochim. Biophys. Acta (BBA) - Gene Regul. Mech. 1809, 360–368. doi: 10.1016/j.bbagrm.2011.04.006 21549230

[B55] Wang X. Jin S. Chang X. Li G. Zhang L. Jin S. (2021). Two interaction proteins between AtPHB6 and AtSOT12 regulate plant salt resistance through ROS signaling. Plant Physiol. Biochem. 169, 70–80. doi: 10.1016/j.plaphy.2021.11.001 34773804

[B56] Wohlgemuth H. Mittelstrass K. Kschieschan S. Bender J. Weigel H. J. Overmyer K.. (2002). Activation of an oxidative burst is a general feature of sensitive plants exposed to the air pollutant ozone. Plant Cell Environ. 25, 717–726. doi: 10.1046/j.1365-3040.2002.00859.x

[B57] Xie M. Hong C. Zhang B. Lowdon R. F. Xing X. Li D.. (2013). DNA hypomethylation within specific transposable element families associates with tissue-specific enhancer landscape. Nat. Genet. 45, 836–841. doi: 10.1038/ng.2649 23708189 PMC3695047

[B58] Xu Q. (2023). The role of DNA demethylase ROS1 in maize kernel development (Hubei, China: Huazhong Agricultural University).

[B59] Yang L. Lang C. Wu Y. Meng D. Yang T. Li D.. (2022). ROS1-mediated decrease in DNA methylation and increase in expression of defense genes and stress response genes in Arabidopsis thaliana due to abiotic stresses. BMC Plant Biol. 22, 104. doi: 10.1186/s12870-022-03473-4 35255815 PMC8903643

[B60] Yang X. Lu X. Chen X. Wang D. Wang J. Wang S.. (2019). Genome-wide identification and expression analysis of DNA demethylase family in cotton. J. Cotton Res. 2, 16. doi: 10.1186/s42397-019-0033-2

[B61] Yu L. Sun Y. Zhang X. Chen M. Wu T. Zhang J.. (2022). ROS1 promotes low temperature-induced anthocyanin accumulation in apple by demethylating the promoter of anthocyanin-associated genes. Horticulture Res. 9, uhac007. doi: 10.1093/hr/uhac007 PMC9123231 35147161

[B62] Zhang J. Xie M. Yu G. Wang D. Xu Z. Liang L.. (2023). CaSPDS, a spermidine synthase gene from pepper (Capsicum annuum L.), plays an important role in response to cold stress. Int. J. Mol. Sci. 24, 5013. doi: 10.3390/ijms24055013 36902443 PMC10003509

[B63] Zhang H.-X. Zhu W.-C. Feng X.-H. Jin J.-H. Wei A.-M. Gong Z.-H. (2020). Transcription factor caSBP12 negatively regulates salt stress tolerance in pepper (Capsicum annuum L.). Int. J. Mol. Sci. 21, 444. doi: 10.3390/ijms21020444 31936712 PMC7013666

[B64] Zhao Y. Xie S. Li X. Wang C. Chen Z. Lai J.. (2014). REPRESSOR OF SILENCING5 encodes a member of the small heat shock protein family and is required for DNA demethylation in arabidopsis. Plant Cell 26, 2660–2675. doi: 10.1105/tpc.114.126730 24920332 PMC4114958

[B65] Zhao F. Zhao T. Deng L. Lv D. Zhang X. Pan X.. (2017). Visualizing the essential role of complete virion assembly machinery in efficient hepatitis C virus cell-to-cell transmission by a viral infection-activated split-intein-mediated reporter system. J. Virol. 91, 10-1128. doi: 10.1128/JVI.01720-16 PMC5215325 27852847

[B66] Zhu H. Xie W. Xu D. Miki D. Tang K. Huang C.-F.. (2018). DNA demethylase ROS1 negatively regulates the imprinting of DOGL4 and seed dormancy in Arabidopsis thaliana. Proc. Natl. Acad. Sci. 115, E9962–E9E70. doi: 10.1073/pnas.1812847115 30266793 PMC6196528

[B67] Zörb C. Geilfus C. M. Dietz K. J. (2019). Salinity and crop yield. Plant Biol. 21, 31–38. doi: 10.1111/plb.12884 30059606

[B68] Zou X. Zhu F. (2022). Origin, evolution and cultivation history of the pepper. Acta Hortic. Sin. 49, 1371–1381. doi: 10.16420/j.issn.0513-353x.2021-0853

[B69] Zulfiqar F. Ashraf M. (2021). Nanoparticles potentially mediate salt stress tolerance in plants. Plant Physiol. Biochem. 160, 257–268. doi: 10.1016/j.plaphy.2021.01.028 33529801

