# Supplementary material for: Functional identification of DNA demethylase gene CaROS1 in pepper (Capsicum annuum L.) involved in salt stress
Source: Front Plant Sci. 2024 May 1;15:1396902. doi: 10.3389/fpls.2024.1396902 (PMC11097670; doi:10.3389/fpls.2024.1396902)
Supplement: Supplementary file 1 [file DataSheet_1.docx]

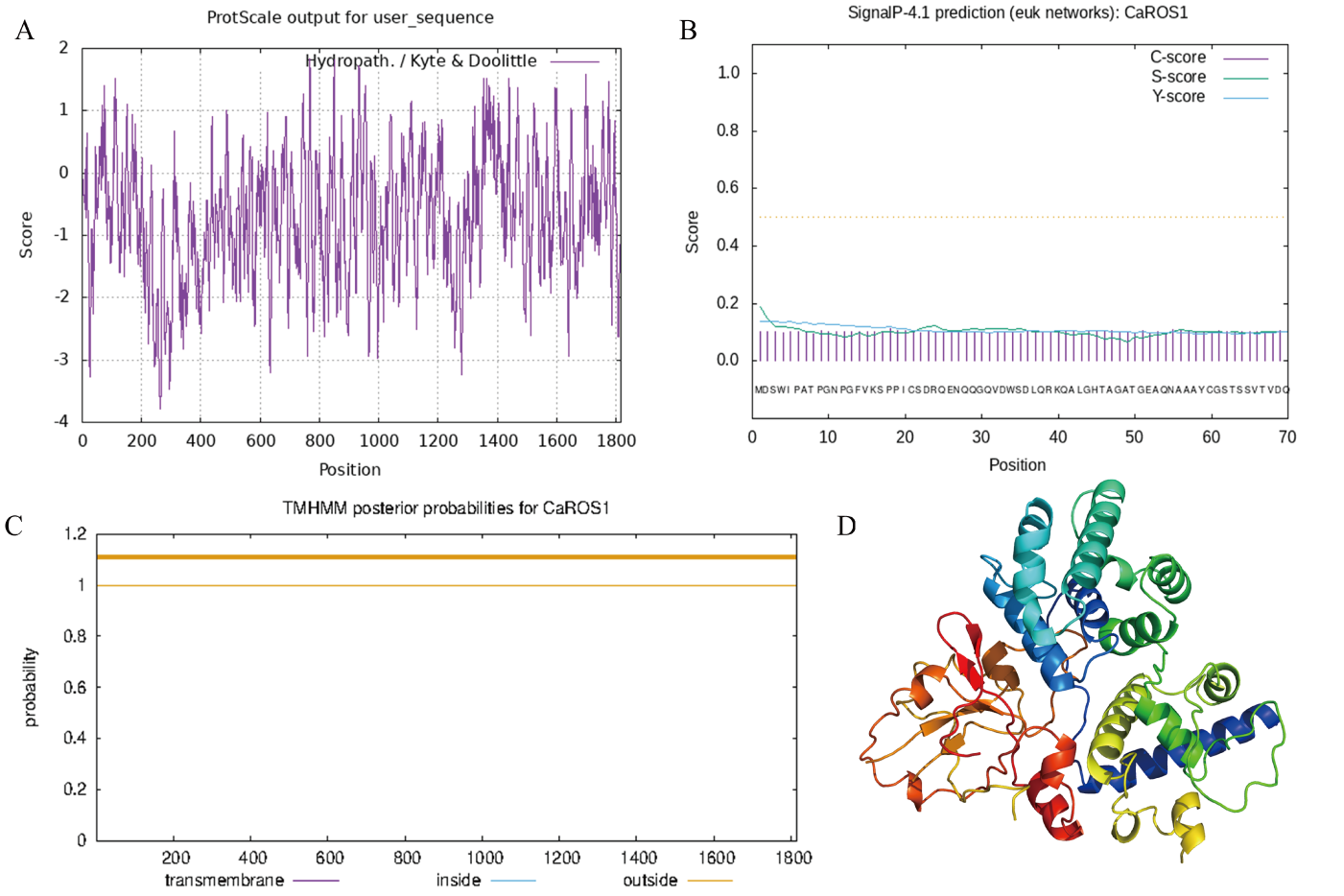


**Figure S1.** Bioinformatics analysis of CaROS1 in pepper. (**A**) Hydrophilicity/hydrophobicity prediction of CaROS1 protein. (**B**) Signal peptide prediction of CaROS1 protein. (**C**) Robability of CaROS1 protein located in/out/across membrane. (**D**) Predicted tertiary structure of CaROS1.


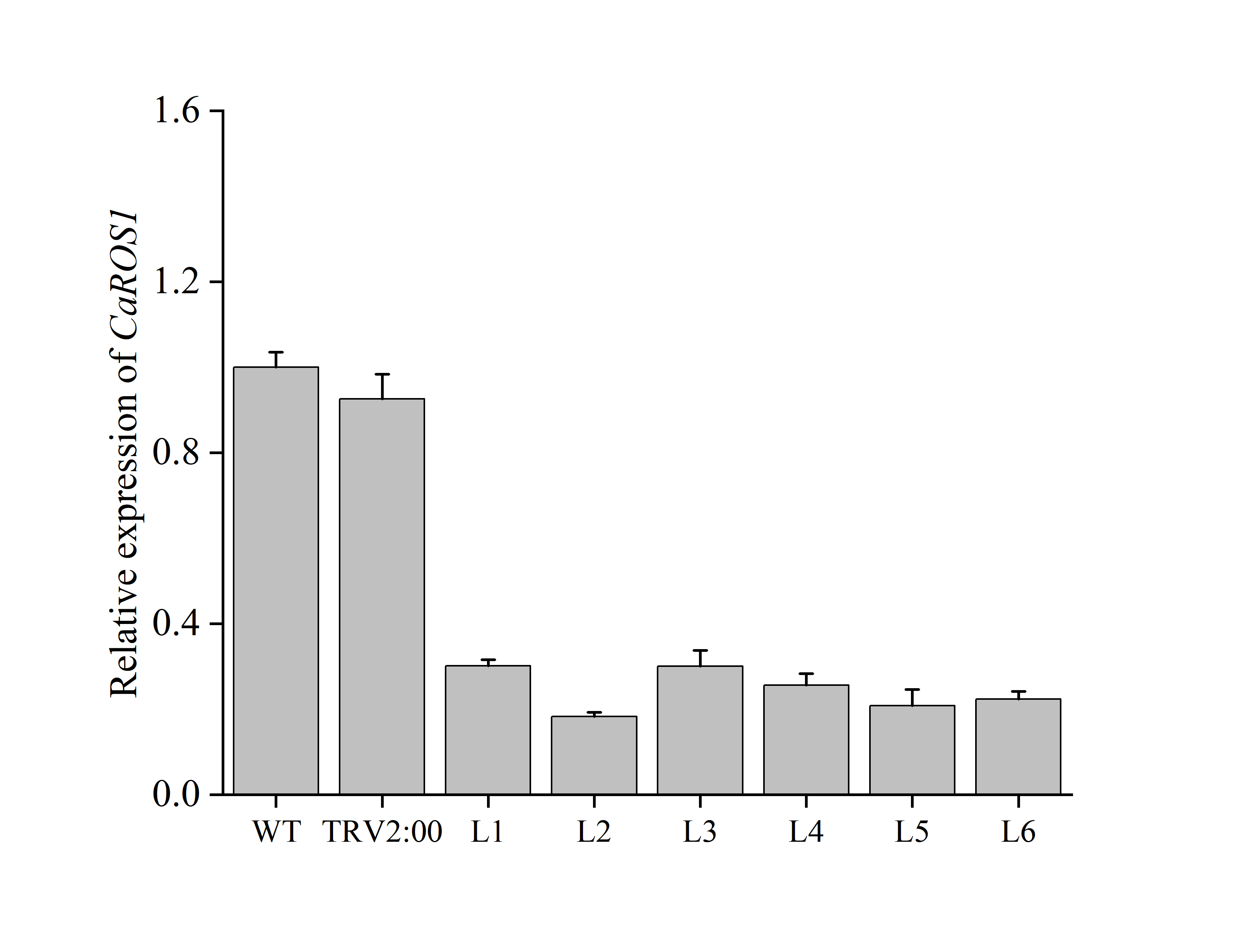


**Figure S2.** Analysis of *CaROS1* expression levels by qRT-PCR after VIGS. WT: Seedlings without bacterial liquid injection; TRV: 00, seedlings inoculated with empty vector; L1~L6: seedlings of *CaROS1*-silenced.


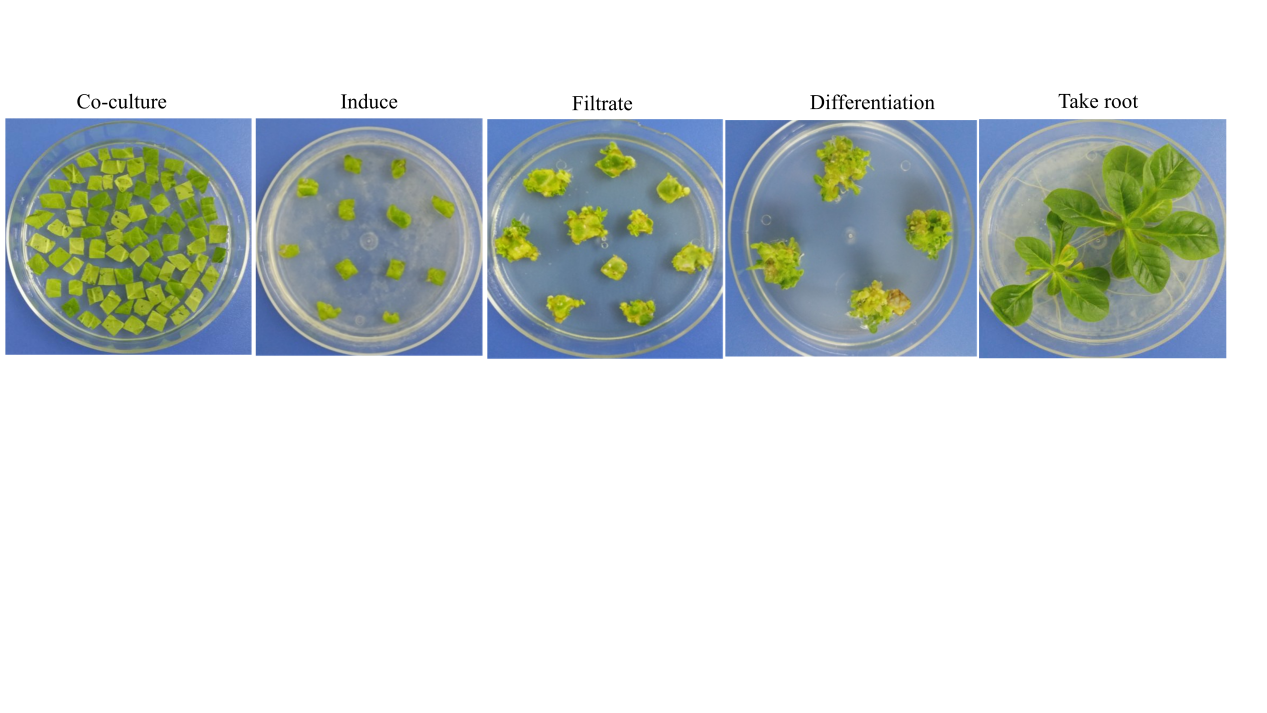


**Figure S3.** Generation of *CaROS1* transgenic tobacco.


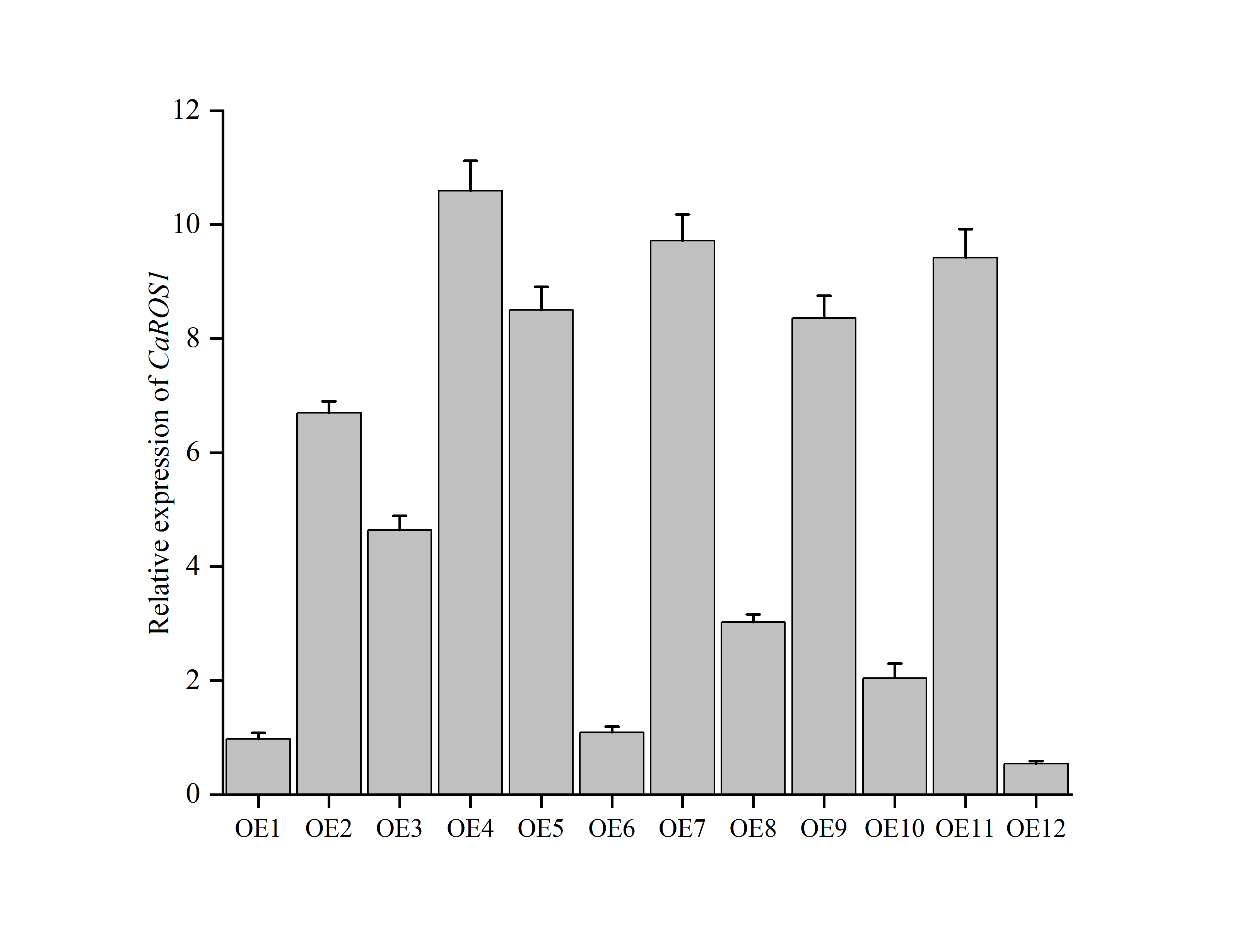


**Figure S4.** Identification and Expression Level analysis of *CaROS1* in transgenic tobacco. Relative expression level of *CaROS1* gene in all transgenic lines. The line with the OE-1 expression of *CaROS1* was used as the control, and was set as 1.
