# Supplementary material for: Functional identification of DNA demethylase gene CaROS1 in pepper (Capsicum annuum L.) involved in salt stress
Source: Front Plant Sci. 2024 May 1;15:1396902. doi: 10.3389/fpls.2024.1396902 (PMC11097670; doi:10.3389/fpls.2024.1396902)
Supplement: Supplementary file 2 [file Table_2.docx]

**Table S1.** Bioinformatics analysis website

| **Website** | **Interlinkage** |
| --- | --- |
| ExPASy | <https://www.expasy.org/protparam/> |
| GSDS | <http://gsds.cbi.pku.edu.cn/> |
| ProtScale | <http://web.expasy.org/protscale> |
| SignaIP 4.1 | <https://services.healthtech.dtu.dk/service.php?SignalP-4.1> |
| TMHMM 2.0 | <https://services.healthtech.dtu.dk/service.php?TMHMM-2.0> |
| SOPMA | <https://npsa-prabi.ibcp.fr/cgi-bin/npsa_automat.pl?page=npsa%20_sopma.html> |
| SWISS-MODEL | <https://swissmodel.expasy.org/interactive> |

**Table S2**. Sequences of the primers used in this study

| **Primer name** | **Primer sequences** |
| --- | --- |
| CaROS1-F  CaROS1-R | agtggtctctgtccagtcctATGGACTCATGGATCCCAGCAA  ggtctcagcagaccacaagtTTAGGAGGCTACTCCTTTGTCTTC |
| EGFP-CaROS1-F  EGFP-CaROS1-R | CATGGTCCTGCTGGAGTTCGTG  ACCGGCAACAGGATTCAATC |
| Q-CaROS1-F  Q-CaROS1-R | GGGGAAACTAACTATGTGC  GATCTCCTCCTTAACCTCT |
| Q-CaActin-F  Q-CaActin-R | GGTGACGAGGCTCAATCCAA  CTCTGGAGCCACACGAAGTT |
| TRV2-CaROS1-F  TRV2-CaROS1-R | agtggtctctgtccagtcctGGCAGCCAAAATCAA  ggtctcagcagaccacaagtTCGGGTTCTTCAATTATA |
| QTRV2-CaROS1-F  QTRV2-CaROS1-R | GGGAGAGTGCCCTTGTTCTT  ACCCGTGTTTCCTTCATGCT |
| QNtActin-F  QNtActin-R | TACTTACTGAAGCACCCTTGAATCC  GATCACGACCAGCAAGATCCAAC |

**Note:** F, forward primer; R, reverse primer; Lower case letters are universal primers.
